# Stem Cell Therapy and Its Significance in HIV Infection

**DOI:** 10.7759/cureus.17507

**Published:** 2021-08-27

**Authors:** Khizer Khalid, Jaskamal Padda, Ransirini Wijeratne Fernando, Krutagni Adwait Mehta, Abdulelah H Almanie, Hussam Al Hennawi, Sandeep Padda, Ayden Charlene Cooper, Gutteridge Jean-Charles

**Affiliations:** 1 Internal Medicine, JC Medical Center, Orlando, USA; 2 Internal Medicine, AdventHealth & Orlando Health Hospital, Orlando, USA

**Keywords:** stem cell therapy, hiv-1, hiv-2, acquired immune deficiency syndrome (aids), cd4+ cell count

## Abstract

Human immunodeficiency virus (HIV) infection is a major global public health issue. Despite this, the only treatment available in mainstay is antiretroviral therapy. This treatment is not curative, it needs to be used lifelong, and there are many issues with compliance and side effects. In recent years, stem cell therapy has shown promising results in HIV management, and it can have a major impact on the future of HIV treatment and prevention. The idea behind anti-HIV hematopoietic stem/progenitor cell (HSPC)-directed gene therapy is to genetically engineer patient-derived (autologous) HSPC to acquire an inherent resistance to HIV infection. Multiple stem-cell-based gene therapy strategies have been suggested that may infer HIV resistance including anti-HIV gene reagents and gene combinatorial strategies giving rise to anti-HIV gene-modified HSPCs. Such stem cells can hamper HIV progression in the body by interrupting key stages of HIV proliferation: viral entry, viral integration, HIV gene expression, etc.Hematopoietic stem cells (HSCs) may also protect leukocytes from being infected. Additionally, genetically engineered HSCs have the ability to continuously produce protected immune cells by prolonged self-renewal that can attack the HIV virus. Therefore, a successful treatment strategy has the potential to control the infection at a steady state and eradicate HIV from patients. This will allow for a potential future benefit with stem cell therapy in HIV treatment.

## Introduction and background

WHO estimated an human immunodeficiency virus (HIV) prevalence of 37.7 million people by the end of 2020, with 680,000 deaths due to HIV-related causes. Globally, HIV has claimed 36.3 million lives so far [1]. The evolution of stem cell research and conduction of applied clinical trials have unveiled the great expectations of stem cell therapy in various medical conditions and applications [2]. The therapeutic clinical potential of stem-cell-based therapy is relatively promising due to the cellular characteristics of stem cells. They are referred to as precursor cells to all types of cells, giving them the ability of regeneration and repair in different types of tissues. There are two types of stem cells. The first is the embryonic stem cells, which originate from the zygote of blastocyst and can generate all subtypes of cells in the body. The second type is the adult stem cells, which can give rise to specific types and forms of cells. In general, stem cell therapy has been applied to variable types of pathological injury processes like cancer and periodontal disorders [3]. Clinical trials involving stem cell therapy have shown significant promising therapeutic capabilities in the treatment of various types of autoimmune, degenerative, and genetic types of disorders [4]. Stem cell therapy has also been implemented in wound-healing therapy. Clinical trials have shown the possibility of complete tissue restoration after treatment [5].

Moreover, it has been implemented that the use of stem cell therapy in HIV patients can augment the process of keeping the viral load in a dormant state with an ability to taper antiretroviral regimens. Surprisingly, this practice brought about substantial improvement in procedure outcome soon after antiretroviral therapy was started. Stem cell therapy involves transplanting stem cells from a donor who has cysteine-cysteine chemokine receptor 5 (CCR5) delta 32 homozygosity to an HIV-infected patient [6]. Another implemented modality is establishing stem-cell-induced HIV resistance via transplanting HIV-fusion competitive inhibitors through stem cells [7]. The aim of using HIV-resistant hematopoietic stem cells (HSCs) is to render the immune system resistant against the HIV infection, which will eventually reduce the possibility of antiviral indefinite therapy [8]. Since HIV type 1 (HIV-1) can remain in a dormant stage in cluster of differentiation 4 positive (CD4+) lymphocytes and macrophages, antiretrovirals carry a limited capacity in complete elimination of the virus. Stem cell transplantation can be considered for the possibility of eliminating the viral genomes from the dormant state in CD4+ lymphocytes and macrophages either by gene editing or cytotoxic antiviral cells [9]. For the HIV virus incorporation into lymphocytes through entry, the process is dependent upon the presence of a functional receptor CXC chemokine receptor 4 (CXCR4) and CCR5. An HIV-infected case has been reported where viral replication was not established after the transplantation of stem cells with modified CCR5Δ32/Δ32 [10]. After the transplant, it was observed that the patient did not experience viral load rebound. In this article, we are proposing a review on stem cell therapy and its use in HIV infection via highlighting the potential impact of the treatment on the disease activity and outcome.

## Review

Human immunodeficiency virus

The HIV epidemic first appeared in 1981, when a new disease syndrome was identified in human populations worldwide, characterized by a deficiency in the immune system [11]. The acquired immune deficiency syndrome (AIDS) appeared to have a marked reduction in CD4+ cell numbers, enhanced B-cell proliferation, and hypergammaglobulinemia [11]. The causative agent of AIDS, later identified as HIV, is a retrovirus bearing a genomic structure of two identical single-stranded ribonucleic acid (RNA) molecules [12]. Human infection with HIV progresses through three stages: acute infection, chronic infection, and AIDS [13]. In the acute infection stage, the circulation has a large viral load, is highly contagious, and may or may not present with flu-like symptoms. In the chronic stage, the viral load is less than the acute stage, with individuals still being contagious but may be asymptomatic. The final stage of AIDS occurs when the CD4+ cell count drops below 200 cells/mm or when opportunistic infections develop [13].

There are two types of HIV isolates that are identified currently, HIV type 1 and HIV type 2 (HIV-2) [12]. The main causative agent of AIDS worldwide is HIV-1, while HIV-2 is confined to some regions of Western and Central Africa. While both viruses can cause AIDS, disease of the central nervous system is mostly seen with HIV-2 infection [12]. Moreover, HIV-2 appears less virulent than HIV-1 and it takes longer to develop AIDS when infected with HIV-2. Although both HIV-1 and HIV-2 types have a similar genetic structural makeup consisting of group-specific antigen (gag), polymerase (pol), and envelope (env) genes, they differ in the organization of their genome.

**Figure 1:**
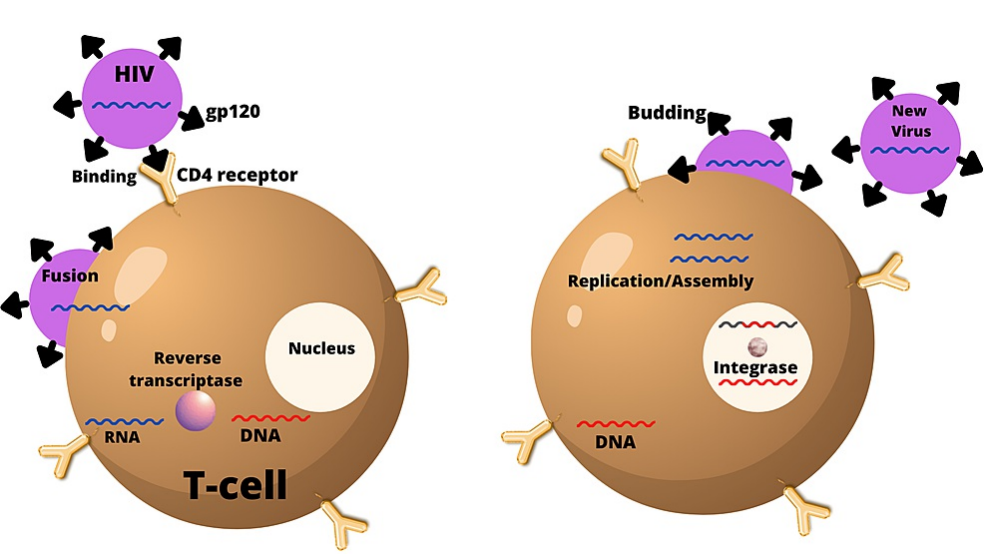
T cell and HIV interaction HIV, human immunodeficiency virus; CD4 receptor, cluster of differentiation 4 receptor; RNA, ribonucleic acid; DNA, deoxyribonucleic acid; gp120, glycoprotein 120 Image created by the author (Jaskamal Padda, MD)

HIV invades the immune cells, CD4+ T cells, and monocytes that leads to a fall in T-cell numbers below a critical level and loss of cell-mediated immunity [14]. HIV infection of T cells occurs via a high-affinity interaction between the glycoprotein (gp120) present in the virion envelope and the CD4 molecule. The virus infects T cells and monocytes by interacting with their corresponding co-receptors, CXCR4 and CCR5. After the virus attaches and enters the host cell, the retrovirus uses its reverse transcriptase to copy its RNA into deoxyribonucleic acid (DNA) [14]. These new DNA copies then leave the host cell and continue to infect other cells in the host (Figure 1) [12,14,15].

How does stem cell therapy work?

Adult stem cells are present in organisms that are postnatal. These cells can present as unipotent or as multipotent stem cells [16]. The dominant locus of adult stem cells is the bone marrow with a higher cellular percentage of multipotent stem cells. These cells have the capacity to augment, differentiate, and generate the entire hematopoietic cell line. Adult stem cells exist in an undifferentiated pattern and are present in all types of tissues. Usually, these cells remain dormant without constant division. The main purpose of adult stem cells is to regenerate and replace cells that are dead, either due to natural or pathological causes. By taking this regenerative ability into consideration, these cells are highly applicable in the therapeutic process of degenerative disorders. As a prominent example, bone marrow transplants usually involve the process of transplanting HSCs into patients suffering from different types of hematological malignancies. Once transplanted, they start to divide and yield healthy types of cells instead of the preexisting cancerous cells [17].

Human mesenchymal stem cells (MSCs), which are considered mesodermal cells, are precursors that differentiate into adipocytes, chondrocytes, and osteoblasts. They are characterized by strong regenerative capacity in all types of tissues [18]. On the other hand, being the less commonly used, MSCs can be isolated and highly regenerated in vitro. Nonclonal stromal cell cultures that are isolated from the bone marrow are the predominant source of MSCs for therapeutic reasons [19]. The biological properties and mode of action of MSCs are not clearly emphasized; their therapeutic properties are usually supported by their ability to differentiate into different cell lineages, production of essential factors that are vital for cell survival, immunomodulation, and their ability to travel to the site of pathology. Given the fact that MSCs can replicate in vivo and in vitro into various types of tissues, they have remarkable abilities to transdifferentiate, as well as produce altered cell types with different characteristics and different lineages [19]. The survival ability of MSCs is also attributed to their ability to produce vital types of cytokines, growth factors, and chemokines. These components can highly augment the process of stem cell adaptation in various micro-environments and repair damaged tissues [19]. Homing mechanism is an adaptive mechanism in which MSCs illustrate the ability to respond to and interact with a diversity of signals in pathophysiological states in different tissues. Although not fully understood, certain types of receptors signaling are involved in cellular migration to the site of injury (homing). The most commonly involved signaling factors are stromal-derived factor 1, hepatocyte growth factor, and receptor tyrosine kinase [19].

Stem cell therapy in treating HIV

Highly active antiretroviral therapy (HAART) for the treatment of HIV has been associated with many complications and compliance issues in patients, which call for a more practical and more effective possible alternative. The development of novel therapeutic strategies addressing a myriad of safety and effectiveness concerns has been the major focus in the HIV/AIDS domain for a life-long remission of the associated infection. To date, the first and only case of HIV cure following allogenic CCR5-deficient bone marrow transplant was reported in 2009. Mr. Timothy Ray Brown, the so-called Berlin patient, an HIV-positive patient, has successfully undergone transplantation of naturally resistant HIV-resistant CCR5 homozygous Δ32/Δ32 bone marrow stem cells following the diagnosis of acute myelogenous leukemia. Interestingly, the Berlin patient demonstrated undetectable HIV viral replication for more than eight years following bone marrow transplantation [6,20,21]. This incident has initiated an endless cascade of interest in promoting long-term HIV cure methods. However, several efforts have been described to replicate similar effectiveness in achieving a second case of HIV cure, but this has not been the case [22,23]. This particular incident has not only demonstrated the promising potential of stem cell therapy in HIV individuals but also revealed possible challenges that may limit successful implementation. Some reported limitations include a scarce number of human leukocyte antigen (HLA)-matched natural CCR5 Δ32/Δ32 allogeneic donors, high-risk bone marrow transplant procedures, and naturally resistant HIV strains. Viral escape mechanisms have also been reported that demonstrate limited effectiveness of the CCR5 knockout scheme to limit HIV replication [24].

The idea behind anti-HIV hematopoietic stem/progenitor cell (HSPC)-directed gene therapy is to genetically engineer patient-derived (autologous) HSPCs to acquire inherent resistance to HIV infection. In theory, HIV-resistant engineered HSPCs have the ability to yield long-life HIV-resistant progenies following one-time transplantation. Multiple stem-cell-based gene therapy strategies have been suggested that may infer HIV resistance including anti-HIV gene reagents and gene combinatorial strategies giving rise to anti-HIV gene-modified HSPCs. Below are some mechanisms postulated for anti-HIV gene-modified HSPCs.

Viral Entry Mechanisms in HIV

Lately, research has demonstrated the development of anti-HIV genes against chemokine receptor CCR5, which has become the target of current research conducting anti-HIV HSPC gene therapy mechanisms [8,25]. The CCR5 co-receptor has been shown to play a vital role in HIV infection. Of note, studies demonstrated no adverse effects in the hematopoietic and immune systems of humans following the inhibition of CCR5 expression, except that this might serve as a conduit to more severe cases of infection mediated by other viruses like West Nile virus encephalitis [8,26]. CCR5 Δ32/Δ32 mutation provides natural resistance to HIV transmission in homozygous acquired gene mutation patients. The heterozygous mutation provides a few years slower infection progression to AIDS compared to wild-type CCR5 individuals [27]. The molecular alteration of ribozyme, RNA interference, clustered regularly interspaced short palindromic repeats/CRISPR-associated protein 9 (CRISPR/Cas9), and zinc finger nuclease (ZFN) gene-editing technologies have been reported as possible CCR5 inhibitor mechanisms for anti-HIV HSPC gene therapy.

Even though CCR5 has become the focus of targeted therapy to limit HIV entry, studies have demonstrated HIV resistance to CCR5 monotherapy following the emergence of X4 tropic HIV infection. Interestingly, X4 tropic HIV viral infection is dependent on the CXCR4 co-receptor for entry. This in fact took place after a successful stem cell transplantation from a homozygous defective HSPC donor in an HIV-infected individual. This case shed light on possible viral escape mutations associated with the X4 HIV tropic form, constituting a major limitation to CCR5 inhibition strategies [24]. The method of CXCR4 knockout strategies has been described in 2003 by Anderson et al., and in 2015 by Schuman et al., via short hairpin RNA (shRNA)/ZFN and CRISPR/Cas9 knockout strategies, respectively [28,29]. While this strategy provided protection for mature CD4+ T cells against X4 tropic HIV infection, nevertheless, Anderson et al. suggested that CXCR4 knockout/down has altered HSPC growth, migration, and transformation into more mature hematopoietic cells since CXCR4 expression was noted to play an essential role in CD34 positive (CD34+) HSPCs in terms of differentiation, and quiescence [28,30]. For this reason, CXCR4 gene alteration modalities should only be limited to mature CD4+ T cells. Lastly, C46 constitutes an extensively studied fusion inhibitor to HIV. Fusion inhibitor C46 was essentially developed from the C-terminal repeat of HIV glycoprotein 41. It works as a fusion inhibitor to the N-terminal hydrophobic α-helix of HIV glycoprotein 41 hindering HIV and cell membrane fusion, thus providing resistance to HIV [31,32]. This particular mechanism has been tested for anti-HIV HSPC-directed gene therapy in non-human recipients [33]. However, resistance mutations emerged in HIV gp120 raising questionable effects of C46 gene alteration in non-human primates [34].

Viral Integration Mechanisms in HIV

Like other viruses, HIV has been shown to integrate and embed into the host DNA genome and acquire a stable environment for replication in infected cells. Studies have shown a desirable effect of HIV inhibition prior to this step in preventing chronic HIV infection. A study conducted by Sakkhachornphop et al. has established that the anti-HIV zinc finger protein (ZFP), which is noted to be attached to a green fluorescent protein (GFP), targets and binds to the 2-long terminal repeat (2LTR). This process occurs during the pre-integration complex before genome integration and with nanomolar affinity [35]. Following the delivery of the 2LTRZFP-GFP trans-gene into various cell lines, it showed decreased HIV genome integration by 50%, and up to 100-fold decrease in HIV capsid protein p24 production. This suggests that 2LTRZFP-GFP trans-gene alters the integration of HIV to immune cells. Collectively, Sakkhachornphop et al. suggested that 2LTRZFP-GFP demonstrates a promising target for use in HIV gene therapy for future studies [35].

Post-Integration Steps of the HIV Life Cycle and Anti-HIV Genes

Different escape mechanisms to early integration steps of HIV following gene inhibitors have been described in the context of CCR5/CXCR4 and other essential molecules, which urged for alternative mechanisms halting the HIV life cycle later. Among these, studies have described the mechanism of targeting the HIV provirus. Ebina et al. utilized a CRISPR/Cas9 system to excise latent HIV provirus DNA with the aid of guide RNAs targeting the HIV LTRs [36]. This resulted in 30% of the cells losing their provirus; however, when they replicated the experiment with primary peripheral blood mononuclear cells, they did not see any provirus excision or indels in the HIV LTR region [37]. The method of targeting proviruses with anti-HIV genes shows effective HIV inhibition; however, an optimized delivery system needs to be developed in order to achieve an HIV cure utilizing this method.

Anti-HIV Genes Directed Against HIV Gene Expression

HIV trans-activating factor (tat) and transactivation response element (TAR) is an HIV transcriptional activator constituent. Tat interaction with TAR promotes the initiation of early stages of gene expression and elongation of HIV transcripts. Strong et al. developed a transcription activator-like effector nuclease that was able to specifically disrupt the conserved sequence of the TAR domain. Results showed a 55%-60% reduction of enhanced GFP cells and up to 22% associated provirus damage in the HeLa cells expressing CD4 and infected with HIV (HeLA-LAV) cell line [38].

Multiple Anti-HIV Gene Inhibition

Similar to the success of HAART therapy targeting different vital steps in HIV replication and infection, this has set the stage for possible strategies that combine different anti-HIV genes simultaneously to efficiently suppress HIV infection and hinder resistant HIV escape mutations [24]. Brake et al. evaluated four different shRNAs targeting tat/regulator of expression of virion proteins (rev), pol, or gag. Results revealed that these four shRNAs provided effective HIV inhibition when used in combination. Furthermore, in a mixed cell population of non-engineered and engineered cells, HIV escape mutants are perceived if only one shRNA is used to protect the cells. Conversely, if three or more different shRNAs are combined, no escape mutants would be detected [39]. Along with other important studies, this approach testified to possible important strategies to limit HIV infection and halt subsequent HIV escape mechanisms.

Protecting HSCs and leukocytes from infection

The human leukocyte cell type includes CD4 T cells, cluster of differentiation 8 (CD8) T cells, a subset of progenitor cells, B cells, eosinophils, neutrophils, basophils, natural killer cells, macrophages/monocytes, microglia, and dendritic cells [40]. During development, human leukocytes express the CD4 molecule on their surface. As HIV co-receptors also express the CD4 molecule, they are susceptible to perturbation or direct infection. In a person infected with HIV, human leukocyte cell types may act as a viral pool. A successful stem cell therapy can protect both the T-cell lineage and myeloid lineage. The treatment approach to an HIV-infected patient is based on whether HSCs may be protected and to keep leukocytes from being infected. The studies regarding this approach are evolving at present. Many modes of gene therapies including shRNAs, ZFN, dominant negative protein, decoy RNAs, intracellular antibodies, and anti-HIV ribozymes have either limited the ability of viruses to divide or make cells less susceptible to infection [41-44].

In a large-scale phase 2 trial on HSC-based gene therapy, researchers made the use of autologous adult HSCs transduced with a retroviral vector containing the trans-activator of transcription and viral protein R (tat-vpr)-specific anti-HIV ribozyme in order to produce cells that are less prone to subsequent infection [45]. Though the effect on the viral load was not significant, cells with vectors in them stayed for a longer duration (>100 weeks in most of the cases) and there were elevated CD4+ T cell counts in the group treated with anti-HIV ribozyme compared to the placebo group. The findings show that this type of stem-cell-based approach can be used in a more conventional and reproducible way. A multipronged RNA-based application has been researched recently targeting CCR5 with ribozyme, targeting tat/rev transcripts with small nuclear RNA, and a decoy of TAR region [42]. This was the first study to use lentiviral-based gene therapy vectors that have the ability to reform both the dividing and non-dividing HSCs. They were less likely to cause cellular transformation in comparison to murine retroviral-based vectors. There was also a finding that vector-containing cells had prolonged hematopoiesis of different cell lines. This strategy was found to be safe, and a lentiviral-based approach seemed to have high potential for the future.

In one of the studies, a German patient who had undergone HSC transplantation from CCR5-/- donor could restrict virus multiplication even without antiretroviral therapy [6]. Based on the finding that people who lack CCR5 expression are relatively immune to infection, scientists are exploring the possibilities to eliminate the expression of CCR5 via RNA interference or via ZFN [46]. However, things are not as easy as such; CCR5 -/- infections have also been reported, limiting the potential efficacy of an approach aimed at CCR5 [47]. Despite this, there is much evidence such as slow development of disease in CCR5 heterozygous correlation of CCR5 levels in sooty mangabeys and lack of disease progression highlighting the significance of this approach [48].

Antiviral immunity through stem cells

One of the key challenges in eradicating HIV is the impaired immune function and incomplete immune recovery that the infection causes. In contrast to most mainstream antiviral therapies, genetically engineered HSCs have the ability to continuously produce protected immune cells by prolonged self-renewal that can attack the HIV virus [49]. Therefore, a successful treatment strategy has the potential to control the infection at a steady state and eradicate HIV from patients eventually by a single or minimal treatment.

Long-term immune responses are launched against pathogens by generating antibodies targeting viruses at the lymphoid follicles, which are organized structures comprising immune cells [49]. These important regions and their defense mechanisms are impaired very early following HIV infection. For example, the lymphoid tissue in the intestines is one of the early sites for viral replication and the formation of viral reservoirs. HIV infection results in severe loss of intestinal mucosal T immune cells and disruption of the intestinal epithelial barrier lining, which leads to a leaking intestine [50]. Although antiretroviral drugs are effective in suppressing viral replication, they are not effective in repairing the damage caused by the virus in the immune system. Antiviral drugs cannot restore the function of the lymphoid follicles that are damaged by HIV infection. Studies have shown that bone-marrow-derived MSCs can alter and remodel damaged mucosal sites with a rapid rise in antibodies and T immune cells that target the virus [50]. Therefore, stem cells had been capable of recovering and restoring the mucosal immunity very early on, even without the use of antiviral drugs, thereby increasing the host’s antiviral response.

## Conclusions

HIV infection has been and continues to be a major healthcare burden globally. The initiation of HAART proved to be effective and remains to be a major targeted therapy against HIV progression despite issues with compliance and side effects. Demonstrated by the first case of HIV infection remission achieved by bone marrow transplant, anti-HIV HPSC-based stem cell therapy and gene engineering have substantiated a potential next-generation approach to fight against HIV infection. Engineering of different anti-HIV genes targeting different steps of HIV infection has been experimented with the aid of advanced molecular modalities. Targeting genes involved in the infection have been experimented in different in vivo and in vitro animal models, which protected HSPCs and subsequent mature cells against HIV infection. The safety and application of anti-HIV gene engineering of HSPCs has been established. Nevertheless, cells have expressed insufficient anti-HIV byproducts in order to achieve therapeutic benefits suggesting that the engraftment of anti-HIV gene engineered HSPC needs to be upgraded for best results. Moreover, unlike the first case of HIV cure following bone marrow transplant indicated for an underlying hematological malignancy, intensive myeloablative protocols prior to transplant will be difficult to implement. Results of these trials will dictate future HIV treatment plans and address safety issues of anti-HIV HSPC-based gene therapy and stem cell transplantation.
